# A Petrified Lens and an Ossified Chorion Occurring in the Same Eyeball*Read at the East Texas Medical Association, Palestine, Texas.

**Published:** 1905-10

**Authors:** Sam. R. Burroughs

**Affiliations:** Buffalo, Texas


### TEXAS MEDICAL JOURNAL.

ESTABLISHED JULY, 1885.

PUBLISHED MONTHLY.—SUBSCRIPTION $1.00 A YEAR.

VOL. XXI. AUSTIN, OCTOBER, 1905. No. 4.

For Texas Medical Journal.

### A Petrified Lens and an Ossified Chorion Occurring in the Same Eyeball.*

BY SAM. R. BURROUGHS, M. D., BUFFALO, TEXAS.

Mr. T. N., farmer, age 23, possessing a stout robust constitution, with no history of rheumatism, tuberculosis or syphilis, in his eighth year was attacked with a severe form of typhoid fever, during convalesence from which, gradual loss of vision in the right eye began, which, in the course of several months, resulted in complete loss of the power to see. During this entire period, in which the tissues of the eye were undergoing changes so detrimental to its subsequent usefulness, not one symptom of inflammation was experienced, but the painless and gradual loss of vision was finally accomplished. Covering a period of fifteen years, extending from childhood to mature manhood, no change whatever occurred in the external appearance of this eye, but on the contrary, it preserved its normal presentation. The ophthalmoscope, doubtless, would have discovered that pathologic changes were developing.

About four or five weeks before application for treatment was made, slight, deep-seated pain was experienced, followed by a congested condition of all the tissues of the globe. This condition gradually developed into a panophthalmitis, with a sympathetic response from the left eye. At this juncture the subject applied for treatment, and on examination, after having studied the history of the case, it was found that tension had reached its extreme limit, and all the tunics of the eye were in a state of congestive inflamma-

*Read at the East Texas Medical Association, Palestine, Texas.

tion, attended with great photophobia and copious lachrymation. In the lower part of the anterior chamber, was discovered a white substance, which at first was thought to be pus, but afterwards proved to be the lens. No critical examination was made to determine the nature of this white body, since the globe had already been condemned to removal.

The patient was informed that the only treatment which would offer any permanent relief from his painful condition, and, at the same time preserve the vision in the left eye, consisted in the extirpation of the globe of the affected eye. To this procedure he at once acceded, and on the 16th day of July, 1903, with the observance of all the aseptic and antiseptic precautions in vogue, the patient was anesthetised by Dr. S. N. Aston, who kindly assisted me, both in the operation and the subsequent dissection, and the globe was removed in its entirety by the usual method. The wound was dressed antiseptically; the patient rapidly recovering, was discharged on the fifth day.

On completing the operation, the globe being held in the forceps, was placed in a basin with the pupil looking upwards, in which position it remained several hours. When we proceeded to the dissection, it was found that the white substance which was discovered lying in the anterior chamber, had disappeared behind the iris as an effect of its specific weight—the cornea, anterior chamber, iris and pupil presenting a clear field and normal appearance.

A careful dissection developed the fact that this white body was a dislocated, petrified lens, which had been forced into the anterior chamber by intraocular pressure; and when this pressure was removed, and the pupillary pole of the axis of the globe placed upwards, it gravitated to the posterior chamber, and penetrated the disorganized vitreous humor. The lens was normal in both form and dimensions, with the exception that its posterior surface was characterized by a succession of shallow, though roughened undulations. That it was an excellent specimen of petrifaction, was fairly proven by its size, form, general appearance, weight and the application of the knife. The vitreous humor was disorganized, and its posterior portion discolored, the discoloration, in all probability, being due to the liberation or displacement of the pigmentary cells of the chorion, in the latter's process of ossification. These pigment cells could have found their way into the vitreous body very easily through the broken down retina. The choroid had been transformed into an osseous network, retaining its normal outline, being convex posteriorly, and concave anteriorly. Small bony plates were discovered here and there throughout the ossific structure. No evidence of the branches of the arteria centralis could be observed microscopically, the dissection being conducted without the aid of the microscope.

There are three points only which I desire to render as my chief reasons for having presented this case for your consideration. First, the fact that I have so far, been unable to find a case reported, wherein the lens had undergone petrifaction, in my limited research into the literature of the subject. Second, it has appeared to be, all along, the consensus of opinion with ophthalmologists, that ossification of the choroid never occurs except in lost, shrunken and collapsed eyes, and if this really is the record, then this case would mark a significant exception. Third, as a contribution to statistics.

Selberg Wells remarks that "formation of true bone is not unfrequently met with on the inner surface of the choroid, in eyes which have undergone atrophy and become shrunken." The same author also says "the shrunken eyeball in which a deposit of bone has taken place, is not unfrequently very painful, both to the touch and spontaneously, and may give rise to sympathetic inflammation."

Knapp declares that true ossification occurs in the eye only as a result of plastic inflammation of the capillary layer of the choroid, while cretification may occur in all the tissues of the eye.

Dr. Edward A. Shumway, of Philadelphia, in his most excellent and instructive contribution to the report of the Committee on Exhibit of Pathologic Preparations, at the Section on Ophthalmology, American Medical Association, New Orleans, May 5-8, 1903, says: "As rare congenital tumors, about twelve cases of subconjunctival osteoma have been reported. These have been situated usually in the supro-external portion of the eyeball. * * * * They represent small pieces of true osseous tissue, with lacunæ, bone corpuscles, etc., and are best explained as the result of a temporary adhesion of the amnion to the eyeball, and the subsequent development of bone in the tissue thus formed." Dr. Shumway's contribution, however, is limited to tumors of the conjunctiva, yet we learn from it the fact that while true osseous tissue is often found in choroid, it may also be found as an osteoma of the conjunctiva.

In conclusion, it may be interesting to know (a) that true osseous tissue in this case, formed in the choroid, without the atrophy, collapse or shrunken condition of the eyeball. (b) That the pathologic facts teach that the lens may assume a state of petrifaction. (c) That ossification and petrifaction may occur coincidentally in the same eyeball. (d) That in similar pathologic conditions, long periods of time may be exempt from any subjective indications of their progress or development.

As a histologic analysis of this case, without doubt, would have been of great value, we can only regret facilities essential for the required dissection, were not at our command.

This report has been very hastily prepared, in a late effort to discharge my duty to the membership of your Society, and no one will more keenly realize its many imperfections than its author, yet, if any feature covered, proves of the least interest or worth to any member present, the fact will more than repay the writer for the little time expended in its preparation.

